# ‘Structure units’ as material genes in cathode materials for lithium-ion batteries

**DOI:** 10.1093/nsr/nwz178

**Published:** 2019-11-08

**Authors:** Jiaxin Zheng, Yaokun Ye, Feng Pan

**Affiliations:** School of Advanced Materials, Peking University, Shenzhen Graduate School, China

**Figure 1.**
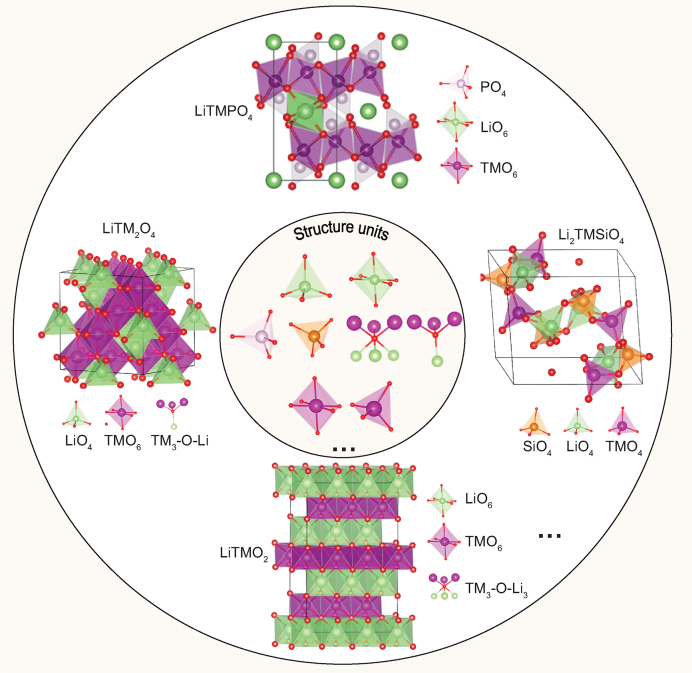
The structure units in the representative cathode materials (olivine LiTMPO_4_, spinel LiTM_2_O_4_, layered LiTMO_2_, Li_2_TMSiO_4_) of LIBs (TM denotes transition metals).

The basic structure unit of crystals is the lattice atom and its coordination environment. They are arranged periodically in certain combinations (e.g. space group) to form crystals. Generally speaking, the bonding interaction and electronic structure in the structure unit determine the intrinsic physical and chemical properties of crystals [1], similar to the key role of genes in life. All the cathode materials for lithium-ion batteries (LIBs) can be formed by combinations of Li, transition metal, and anion structure units (Fig. 1). For example, in olivine polyanion LiFePO_4_ frameworks, FeO_6_ octahedrons with Fe–O coordination bonds are connected by sharing O corners to form a 2D network in the *bc* plane; PO_4_ tetrahedrons with strong P–O covalent bonds are physically separated to connect adjunct FeO_6_ planes; and LiO_6_ octahedrons with Li–O ionic bands are connected by sharing O corners to form a 1D channel on the *b-*axis for Li-ion diffusion. Studying the physical and chemical properties of cathode materials from the perspective of the structure unit will lead to a better and deeper understanding of the intrinsic electrochemical properties and provide guidance for the rational design of cathode materials with high performance from the atomic level, which has attracted increasing attention. Advanced experimental characterizations that are usually conducted to disclose the structure–performance relationship include time-resolved X-ray diffraction, neutron powder diffraction, X-ray absorption, mass spectroscopy, and high-resolution transmission electron microscopy. However, even with these experimental tools it is still difficult to observe the structure units directly, and studying the relationship between structure units and electrochemical properties relies mainly on theoretical simulations, such as *ab initio* calculations, classical and *ab initio* molecular dynamics simulations, and the tight binding method.

**Figure 2.**
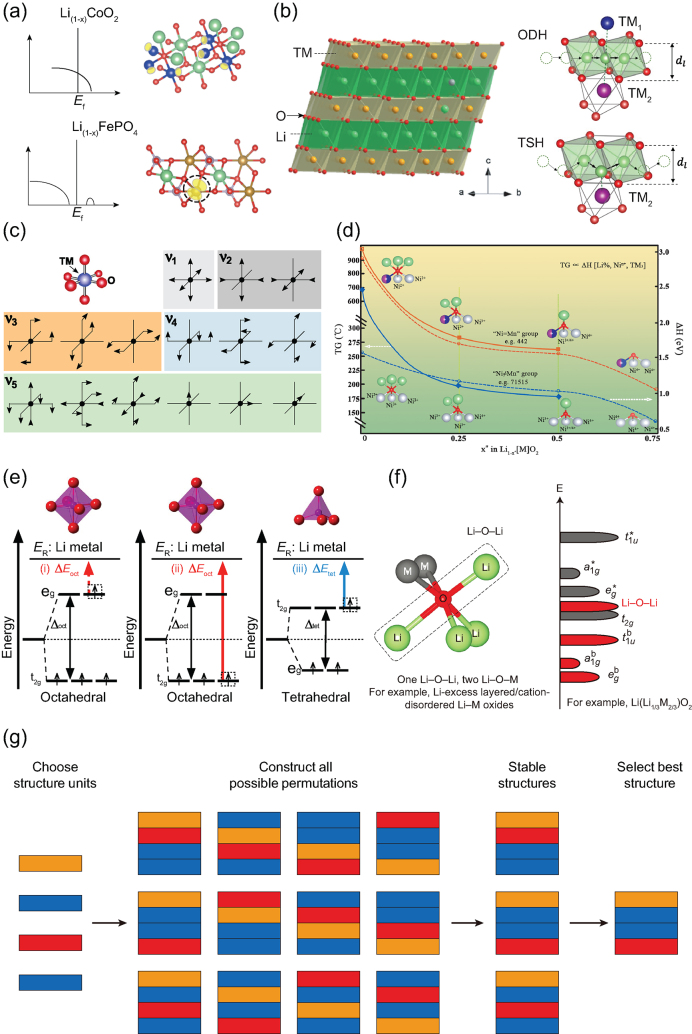
(a) Illustration of density of states and spin density (yellow) around TM atoms after delithiation with *x* content in LiCoO_2_ and LiFePO_4_, respectively. (b) The ODH (oxygen dumbbell hopping) and TSH (tetrahedral site hopping) types of the Li-ion diffusion pathways (right panel) in an α-NaFeO_2_-type structure of layered cathode materials [5]. (c) Different distortion modes for an octahedral TM unit [6]. (d) A global view of the tuning of thermal stability in layered NMC materials during delithiation [8]. (e) Energy levels of the Mn *d* orbitals in octahedral and tetrahedral coordinations [12]. (f) Li–O–Li O 2*p* non-bonding orbitals near the Fermi level induced by Li excess in oxygen structure units (TM_3_–O–Li_3_) [14]. (g) A schematic outlining the computational approach of searching for possible structures arising from structure units. Different color modules denote different structure units [1].

Nearly all the cathode materials of LIBs are semiconductors, and the electronic conductivity relies on the *d* orbital electrons of the transition metals (TM). For example, the diffusion of electrons into and out of LiFePO_4_ relies on the FeO_6_ 2D framework. The coordination environment of TM in TM units (TMO*_x_*) determines the energy and spatial distribution of *d* orbital electrons and thus affects the electronic conductivity directly. For example, the *d* electrons of Co in CoO_6_ in LiCoO_2_ will become delocalized during delithiation (Fig. 2a), and the semiconducting phase transforms to a metallic phase [2].

By contrast, the *d* electrons of Fe in LiFePO_4_ and Li_2_FeSiO_4_ become more localized during delithiation, and the localized electronic state will be coupled to the local atomic lattice distortion to form polarons (Fig. 2a), which is detrimental to the electronic conductivity and finally affects the charge/discharge rate performance [3,4]. The Li-ion transport in cathode materials is also closely correlated with the structure units and their arrangements. For example, the Li ions in layered LiNi*_x_*Mn*_y_*Co*_z_*O_2_ (NMC) diffuse from one octahedral site (LiO_6_) to another octahedral site, passing a gate site controlled by TM ions with one or two octahedral coordination (Fig. 2b) [5]. As a result, the migration of Li ions will be directly influenced by the valence state of TM ions and the size of TMO_6_ and LiO_6_/LiO_4_. Using the above structure unit models, the predicted trend for the variation of the Li-ion diffusion coefficient with Ni content in NMC materials was verified by the experimental galvanostatic intermittent titration technique (GITT) measured results [5].

The structural stability of cathode materials is reported to be related to the structure units. For example, in LiFePO_4_ crystal structure, the PO_4_ tetrahedrons with strong P–O covalence act as joints to connect adjunct FeO_6_ planes to establish the outstanding structural stability during electrochemical cycling. The distortion of TMO_6_ in layered cathode materials during delithiation is the origin of structure disordering and phase transformation of crystal structures (Fig. 2c) [6,7]. Besides the structural stability, the thermal stability of cathode materials also depends on the structure units. Using *ab initio* calculations, we have previously proved that the oxygen structural units (TM_3_–O–Li*_x_*) of lattice oxygen in layered cathode materials determine the thermal stability [8], which can be tuned by changing the TM types and the number of Li ions and modulating their positions in TM_3_–O–Li*_x_* (Fig. 2d). For example, the exchange of Ni and Li in Ni-rich NMC materials would form 180° Ni–O–Ni super-exchange chains in TM_3_–O–Li*_x_*. An O ion between spin-parallel Ni forms σ-bonding with one Ni ion, and an O ion between spin-antiparallel Ni forms π-bonding. This would enhance the thermal stability for Ni-rich NMC materials [7,9]. The theoretically predicted thermal stabilities of NMC materials based on the above TM_3_–O–Li*_x_* unit models agree well with previous and our experimental measurements (*in situ* time-resolved X-ray diffraction and thermal gravimetric analysis) [8,10]. Our recent experimental work combined with theoretical calculations reported that substitution of one-third NiO_6_ with SbO_6_ in NaNiO_2_ to construct a highly ordered (NiO_6_)_6_-ring superstructure within the TM layers would enhance both the structural stability and thermal stability [11].

Most cathode materials are so-called charge-transfer materials, and the charge-transfer process is correlated with the capacity and voltage of cathode materials. TM ions were previously regarded as the sole sources of electrochemical activity in an intercalation cathode to provide the charge-compensating electrons after Li-ion extraction. The energy needed to oxidize TM*^n^*^+^ to TM^(^*^n^*^+ 1)+^ is determined by the energy levels of the TM *d* orbitals in TM units. For example, the energy required to remove an electron from the *t*_2g_ levels of octahedrally coordinated Mn^4+^ is significantly larger than that needed to remove an electron from the *t*_2g_ levels of tetrahedrally coordinated Mn^4+^ (Fig. 2e) [12]. Recent observations have brought the above picture into question and argued that oxygen ions in oxide cathodes may also participate in the redox reaction [13]. With the top valence bands contributed by transition metal–ligand hybridization, it is not very surprising that the oxygen O 2*p* states close to the Fermi level facilitate reversible oxygen redox. Using DFT calculations with the hybrid functional HSE06 (which could correct self-interaction error to some extent), Ceder *et al.* claimed that the oxygen redox in Li-rich layered cathode materials originates from the Li excess in oxygen structure units (TM_3_–O–Li_3_) on the promotion of Li–O–Li O 2*p* non-bonding orbitals at the Fermi level (Fig. 2f) [14]. They further experimentally reported that using F substitution of O in Ni units in Li-rich layered cathode materials can lower Ni^3+^/Ni^4+^ to the Ni^2+^ state, which not only increases the Ni redox reservoir but also prevents the compound from utilizing too much oxygen redox that can trigger oxygen loss [15]. A very recent important theoretical work proved that in Li-rich transition metal oxides, the reversibility of the anionic capacity is limited to a critical number of O holes per oxygen in TMO_6_ units [16].

It should be noted that a synergetic effect of various units also exists. For example, the chemical potential for Li-ion storage in electrode materials at a certain temperature and pressure is determined by the synergetic effect between the kind of redox couples for TM units and the ionic bonding interaction in Li-ion units [17]. As discussed above, the migration of Li ions is also directly influenced by a synergetic effect between TM units (TMO*_x_*) and Li-ion units (LiO*_x_*).

In summary, the structure units in cathode materials can be regarded as material genes, which determine the electronic conductivity, lithium-ion transport, structure and thermal stability, and charge-transfer properties. Understanding the correlation between structure units and physical and chemical properties pushes the traditional electrochemistry in cathode materials for LIBs from a bulk/surface-based interpretation to a more local chemical-bonding interpretation, where the local chemical coordination and local molecular orbitals dominate the physical and chemical properties. This provides easy and direct guidance to improve the electrochemical performance of cathode materials or design high-performance cathode materials by tuning the material genes (like genetic engineering in biology). One way is to tune the material genes directly. For example, substitution of TM and O with other elements in TMO*_x_* units or breaking the symmetry of TMO*_x_* by distortion or pressure would be effective ways to tune the electronic structure in cathode materials. The Li-ion diffusion can be tuned by substitution of TM with different valance state metals in TMO*_x_* units and tuning the sizes and arrangements of TMO*_x_* units and LiO*_x_* units. The other way is to search for possible structures with high performance arising from the material genes (Fig. 2g). First, a set of structure units must be chosen, and the number of times that each structure unit appears within a single repeated unit cell is selected. Next, all the possible permutations of structure units are searched through with the use of methods such as evolutionary algorithms, and the number of candidate structures is reduced by removing duplicates of the same arrangements. Finally, the relative stabilities of the left candidates are evaluated quantitatively by using *ab initio* calculations, and those final theoretically predicted stable structures are ranked according to some suitable selection criteria (e.g., energy density, stability, and rate performance) to give the most promising candidates for experimental synthesis.
